# Effect of ozone and diode laser (635 nm) in reducing orthodontic pain in the maxillary arch—a randomized clinical controlled trial

**DOI:** 10.1007/s10103-019-02896-0

**Published:** 2019-11-05

**Authors:** Jacek Matys, Elżbieta Jaszczak, Rafał Flieger, Katarzyna Kostrzewska-Kaminiarz, Kinga Grzech-Leśniak, Marzena Dominiak

**Affiliations:** 1 Dental Surgery Department, Medical University, Wrocław, Poland; 2 Private Dental Practice, Wschowa, Poland; 3 Private Dental Practice, Kościan, Poland

**Keywords:** Biostimulation, Crowding, Fixed appliance, Photobiomodulation, Teeth

## Abstract

The effect of ozone, diode laser irradiation, and presence of teeth crowding/spacing on pain perception in orthodontic patient was tested. Overall, 76 patients [55 women and 21 men; age 35.1(6.4) years] who met the inclusion criteria participated in the study. Immediately after fixed orthodontic appliance placement, the patients were exposed to a pain relief treatment (one single session) using either 635-nm diode laser (SmartM, Lasotronix, Warsaw, Poland) or ozone therapy (OzoneDTA, Apoza, New Taipei City, Taiwan) by placing the handpieces in the area of each teeth apex and interdental papillae, from the maxillary right first molar to the maxillary left first molar. Subjects were divided into three groups: control group (G1, *n* = 26), ozone (G2, *n* = 26, exposed to ozone therapy, generator probe type 3, working time per point 5 s, 23 points, application time 1 min and 55 s), and laser group (G3, *n* = 25, exposed to continuous mode diode laser, 400 mW, handpiece diameter 8 mm, spot area 0.5024 cm^2^, power density per second 1.59 W/cm^2^, dose 2 J per point, time: 5 s per point, 23 points, total energy per session 46 J, application time 1 min and 55 s). The level of teeth crowding was assessed using the Lundstrom indicator. The patients received a questionnaire for pain assessment (the Numeric Rating Scale, NRS-11, grade level 0–10) and recorded at 7 time points (1 h, 6 h, and 1, 2, 3, 4, and 5 days ) after the fixed orthodontic appliance placement. The mean pain values for the diode laser, ozone, and control group were 3.6 (1.31) (95% CI, 2.95–4.25), 5.25 (3.37) (95% CI, 3.52–6.98), and 5.75 (2.40) (95% CI, 4.69–6.81), respectively. We observed lower pain values in the diode laser group compared to the control group (*p* = 0.0237). The use of ozone in this study did not result in significant pain reduction in comparison to control (*p* = 0.8040) and laser groups (*p* = 0.1029). There were no differences in pain perception between patients with crowded teeth and non-crowded teeth in each group (G1, *p* = 0.66, G2, *p* = 0.86, G3, *p* = 0.24). The use of 635-nm diode laser led to decreased pain perception; however, ozone and presence of teeth crowding/spacing did not affect the pain perception in orthodontic patients during the first 5 days after the fixed orthodontic appliance placement.

## Introduction

The most frequent side effects after fixed orthodontics appliances placement are pain, discomfort, and sensitivity [1–5]. The range of 87 to 95% patients complains about pain after application of orthodontic forces, particularly during the first 24 h, whereas 39 to 49% of them experience discomfort at every stage of treatment or when taking off orthodontics appliances [1–6]. Pain is a common reason which discourages patients from receiving orthodontic treatment [1–3, 7]. Patients’ age, pain threshold, motivation, negative dental experiences, and magnitude of orthodontic force are responsible for feeling pain [7]. Discomfort usually begins 2 to 4 h after the application of force and increases during 24 h and gradually disappears in the next 7 days [3, 8–10]. The pain from increased pressure results in ischemia, inflammation, and edema in the squeezed periodontal ligament (PDL) [11].

There are pharmacological and non-pharmacological methods of pain relief during orthodontic treatment. The most commonly prescribed painkillers in orthodontics are non-steroidal anti-inflammatory drugs (NSAIDs). They were considered the most effective form of pain relief [8]. Unfortunately, they have a lot of side effects, such as gastrointestinal discomfort, thrombocytopenia, skin rashes, hypertension, and headaches [12].

Non-pharmacological methods of relieving pain caused by orthodontic forces include biting wafers, laser therapy, and ozone therapy [13, 14]. Ozone is a gas which has bactericidal, virucidal, and fungicidal properties [15, 16].

A fundamental effect of ozone is improved oxygenation of cells. Furthermore, ozone plasma application activates blood circulation, increases hemoglobin concentration, enhances diapedesis and phagocytosis during inflammatory response, and stimulates all antimicrobial biologic reactions [17]. The reaction of fatty acid peroxidation increases the elasticity of the erythrocyte cell membrane. Thanks to this, cells pass more easily through the capillaries, which significantly improves the metabolism of tissues [17].

The wide range of laser employment in orthodontics includes, e.g., soft tissue surgery [18, 19], hard tissue surgery [20], orthodontic mini-implants insertion [21], accelerating of tooth movement [20], and bracket debonding [22]. Low-level laser therapy (LLLT) is adopted for the relief of pain in orthodontic treatment [23]. The mechanism of action of this device is based on the reaction of sub-cellular photoreceptors to the light generated by the laser. Stimulation of these receptors causes an increase in metabolic processes by affecting the electron transport chain, respiratory chain, and oxidation [24]. LLLT causes dilation of blood vessels and the induction of mast cell degranulation, with the release of pro-inflammatory substances to accelerate tissue healing [23]. The effect of laser therapy on neurons results in stabilization of the membrane potential, which retards the activation of the pain signal [25, 26].

In this study, we examine how a diode laser at a wavelength of 635 nm and an ozone therapy affect the patients’ pain sensations during orthodontic treatment depending on teeth crowding.

## Materials and methods

The trial was designed as a randomized and controlled test. Informed consent was obtained from all participating subjects. Approval was obtained from the Local Ethics Committee of the Faculty of Dentistry, Wrocław Medical University (KB-546/2018).

### Subjects

**Fig. 1:**
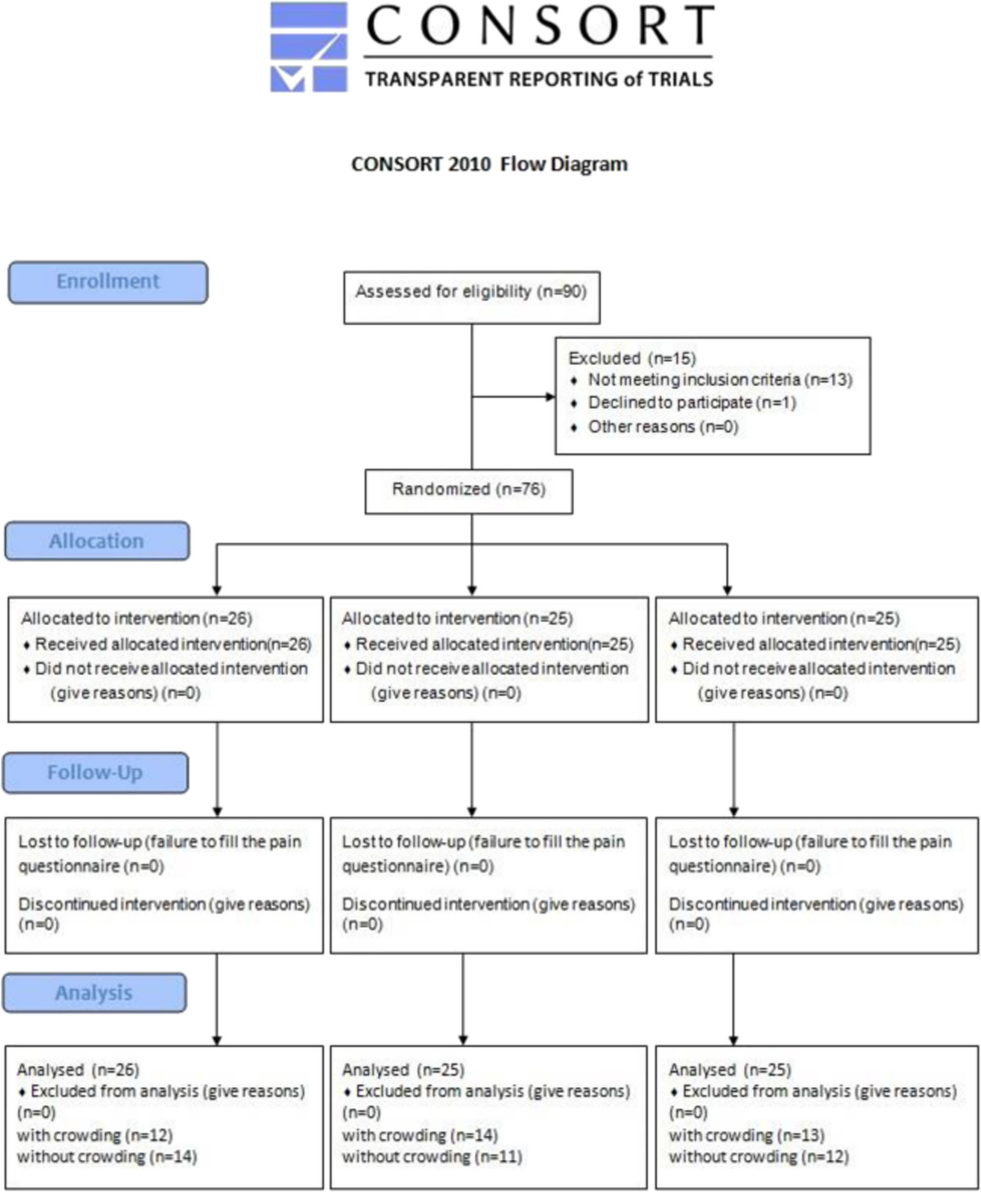
Flowchart of the patients selected for the research according to CONSORT 2010 indications

The research concerned patients of NZOZ Ka-dent (a private healthcare institute) in Wschowa, undergoing orthodontic treatment. The study involved 90 patients (64 women and 26 men; age: 32.6 ± 8.7 years); however, the number of patients who met the inclusion criteria and agreed to participate in this study was 76 (55 women and 21 men; age: 35.1 ± 6.4 years) (Fig. 1).

### The patients selected for the study

All the patients

1. were treated for the first time using a fixed orthodontic appliance
2. had no systemic diseases
3. were not using anti-inflammatory drugs
4. had not used antibiotics in the previous 24 months
5. were non-smokers
6. had fully erupted permanent teeth
7. had no chronic or neural pain
8. had undergone hygienist treatment before the clinical trial

Before the experiments, the experimental protocol and possible side effects were explained to the patients, and their informed consent was obtained.

### Treatment procedure and pain evaluation

**Fig. 2:**
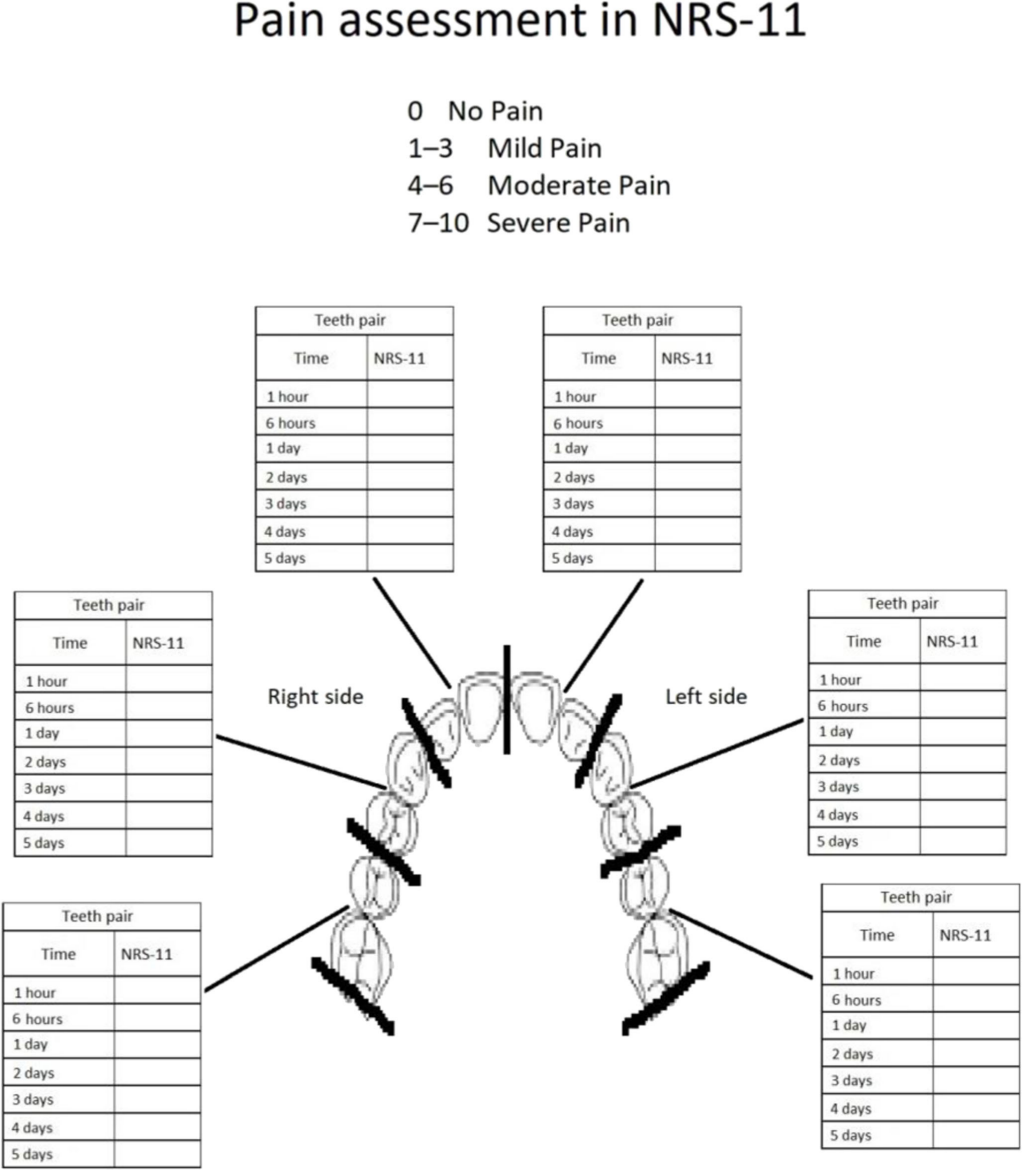
The questionnaire for individual pain assessment on the numeric rating scale (NRS)

Immediately after orthodontic fixed appliance placement, the patients underwent a pain relief treatment using 635-nm diode laser (Smart M, Lasotronix, Poland) or an ozone generator (OzoneDTA; Apoza, Taiwan, ROC) with an intraoral probe by placing the handpieces in the area of each tooth apex and interdental papillae in the maxilla. The patients received a questionnaire for individual pain assessment (the numeric rating scale, NRS-11, grade level 0-10) measured at seven time points: 1 h, 6 h, 1 day, 2 days, 3 days, 4 days, and 5 days after the orthodontic appliance placement. The NRS-11 scale consists of conscious, subjective assessment of the pain experienced; therefore, it is used in the case of patients over 10 years old. A rating of 0 signifies no pain, 1–3 represents mild pain, 4–6 moderate pain, and 7–10 severe pain (Fig. 2).

### Orthodontic treatment

Orthodontic fixed appliances (Legend Mini, GC Orthodontics, USA) that were used in the treatment of our patients had the following prescriptions: MBT, 0.018 slot. As initial arches, NiTI 0.014 was used (Atlas, Prolinx Gmbh, Germany).

### Study groups

*Group 1* (*n = 26*, control): no pain treatment after orthodontic appliance placement.

*Group 2* (*n = 25*): ozone generator OzoneDTA (Apoza, New Taipei City, Taiwan) with the following fixed operation parameters; probe type 3, time per point 5 s; mode: contact, application on each tooth apex area and interdental papillae area, from the first left molar to the first right molar in the maxilla (23 points); total time of ozone application was 1 min and 55 s (one single session).

*Group 3* (*n = 25*): diode laser SmartM (Lasotronix, Warsaw, Poland) at 635-nm wavelength with bio-modulating handpiece with the following set parameters: output power 400 mW, handpiece diameter 8 mm, spot area 0.5024 cm^2^, power density per second 1.59 W/cm^2^, continuous mode, dose 2 J per point, time 5 s per point, 23 points (irradiation on each tooth apex area and interdental papillae area from the maxillary right first molar to the maxillary left first molar), total energy per session 46 J, total time of the laser application was 1 min and 55 s (one single session). The diode laser was used in contact mode with soft tissue only immediately after the orthodontic appliance placement.

The subject allocation for these three groups was conducted using Random Allocation Software (University of Medical Science, Isfahan, Iran).

### Teeth crowding assessment

**Fig. 3:**
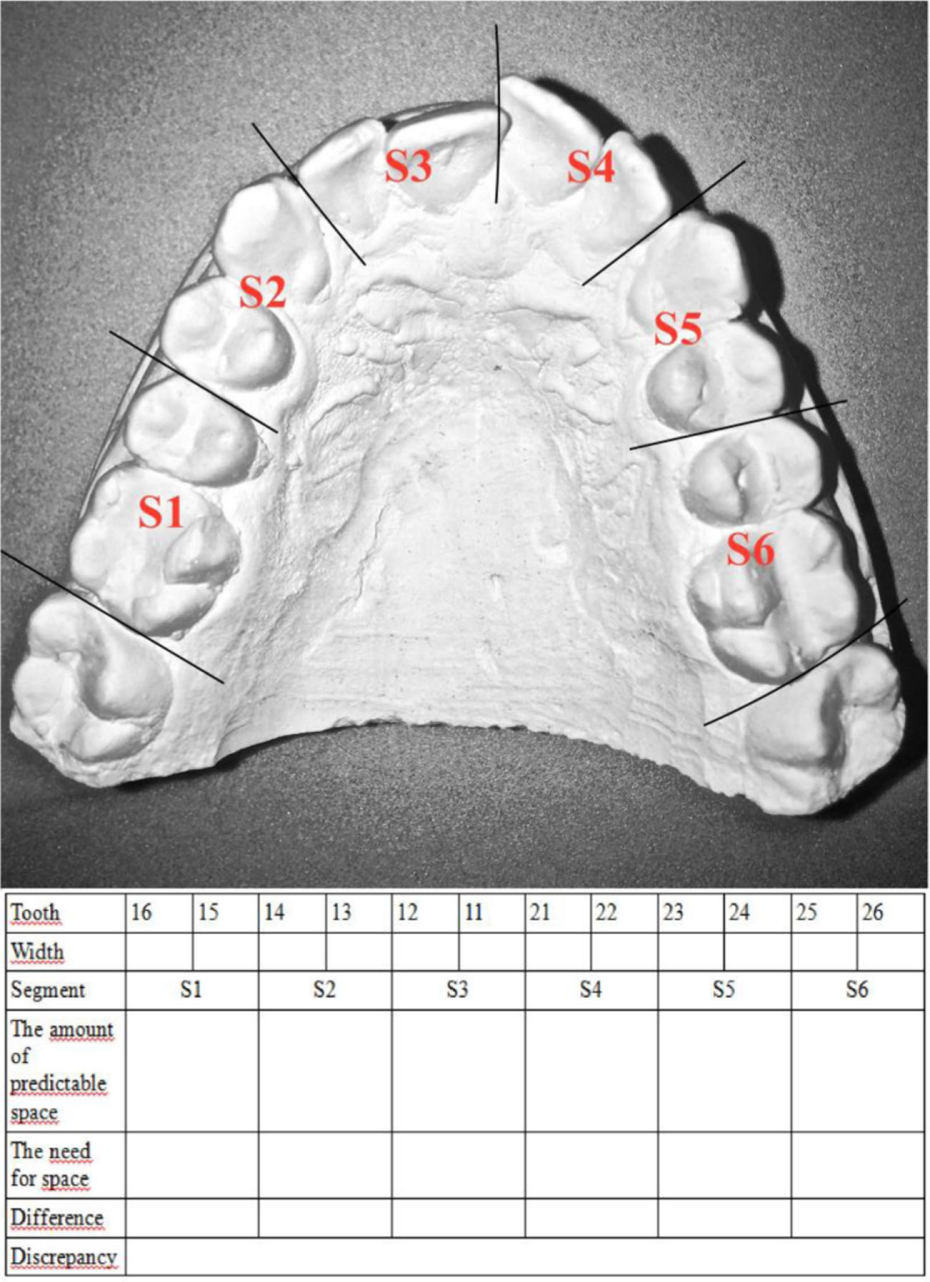
Segments (teeth pairs) in the measurement of crowding for Lundstrom analysis

Patients were qualified according to the level of teeth crowding based on the Lundstrom indicator. The upper dental arch was divided into six segments: S1–S6 (including the first molars). Each segment included a pair of teeth. The length of each segment (between the tangent points of the tooth pairs) and the mesiodistal width of 12 teeth were measured; the measurement result was entered into the table. The sum of the widths of each pair of teeth was determined as the amount of space needed in the dental arch. The differences showed excess or insufficient space in specific segments. By adding the value of S1–S6 differences, information about the size of the upper dental arch deviation from the actual tooth size was obtained. The patients in each group were divided into subjects with (Lundstrom indicator < 0) or without (Lundstrom indicator ≥ 0) teeth crowding (Fig. 3).

### Statistical analysis

All data were subject to statistical analyses with Statistica 12 software (StatSoft, Krakow, Poland). The differences in the mean pain value between the groups were analyzed according to the ANOVA test followed by Tukey’s post hoc test. The differences in the pain related to teeth crowding were assessed using the Mann-Whitney *U* test. Values below *p* = 0.05 were considered to be significant.

## Results

### Evaluation of the highest pain value obtained in the study

The mean pain values on the NRS-11 for the diode laser, ozone, and control group were 3.6 ± 1.31 (95% CI, 2.95–4.25), 5.25 ± 3.37 (95% CI, 3.52–6.98) and 5.75 ± 2.40 (95% CI, 4.69–6.81) respectively. The statistical analysis of the NRS-11 scores revealed significantly lower pain values in the diode laser group in contrast to the control group (*p* = 0.0237). Furthermore, the use of ozone in the research resulted in a lack of pain reduction in contrast to the control subjects (Table 1).

**Table 1:** The mean pain value on the NRS-11 scale for the diode laser, ozone, and control group

Study groups	Number	NRS-11—means	Std. dev.	95% CI	*p* value
Control group (G1)	26	5.75	2.40	4.69–6.81	G1 vs G2 *p* = 0.8040 G1 vs G3 *p* = 0.0237 G2 vs G3 *p* = 0.1029
Ozone group (G2)	25	5.25	3.37	3.52–6.98	
Laser group (G3)	25	3.60	1.31	2.95–4.25	
All groups	76	4.87	2.63	4.17–5.57	

95% CI (confidence interval)

### Teeth crowding did not result in higher mean pain rate

**Fig. 4:**
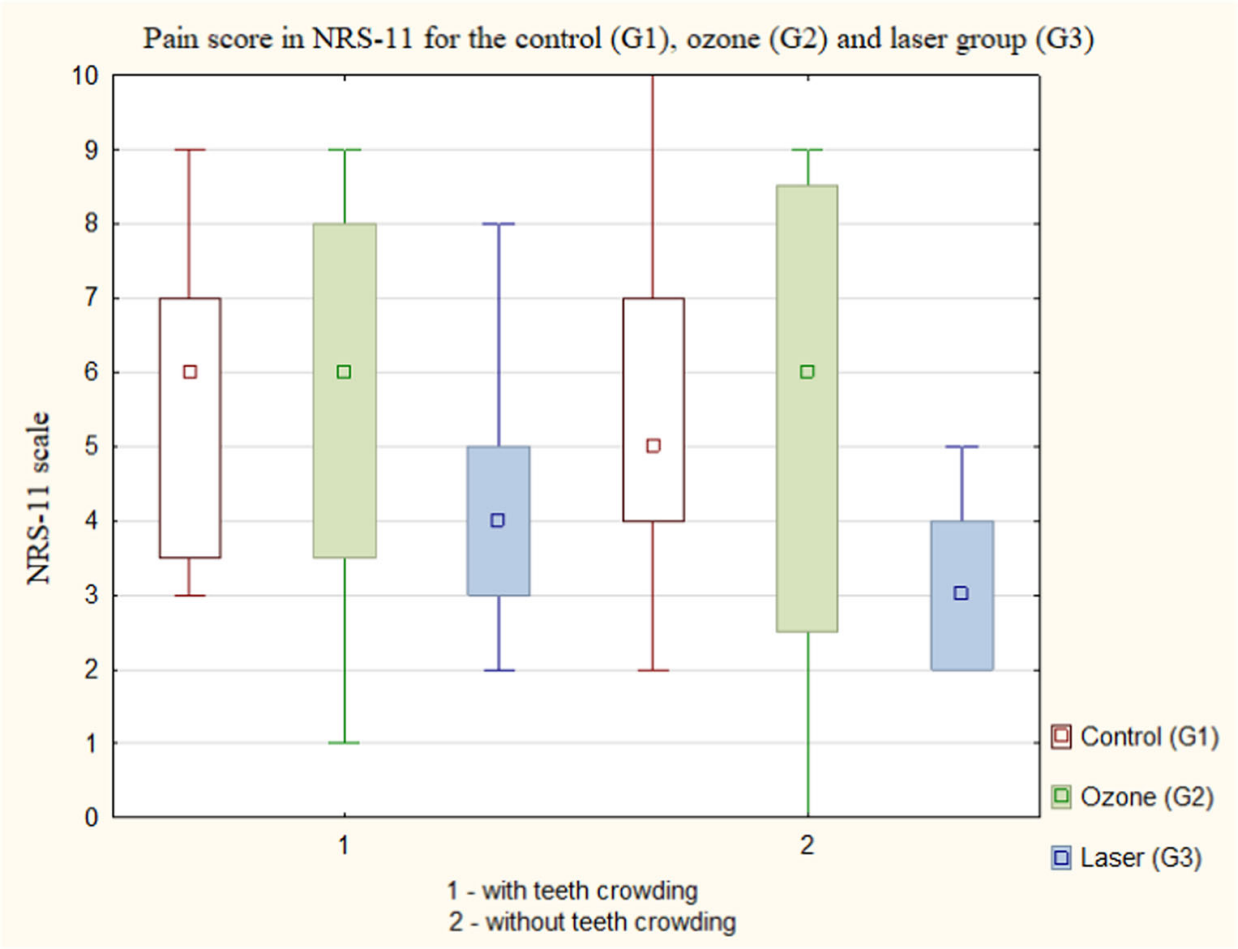
Pain score in NRS-11 for each group with or without teeth crowding

In the present study, we assessed the pain rate for teeth pairs with or without crowding measured by a caliper. The results of this study rejected the hypothesis that teeth crowding caused higher pain rate measured with the NRS-11 scale as compared with non-crowding teeth pairs in all groups: G1 (*p* = 0.66), G2 (*p* = 0.86), G3 (*p* = 0.24) (Fig. 4).

### The mean highest pain score was found 24 h after orthodontic appliance placement

**Fig. 5:**
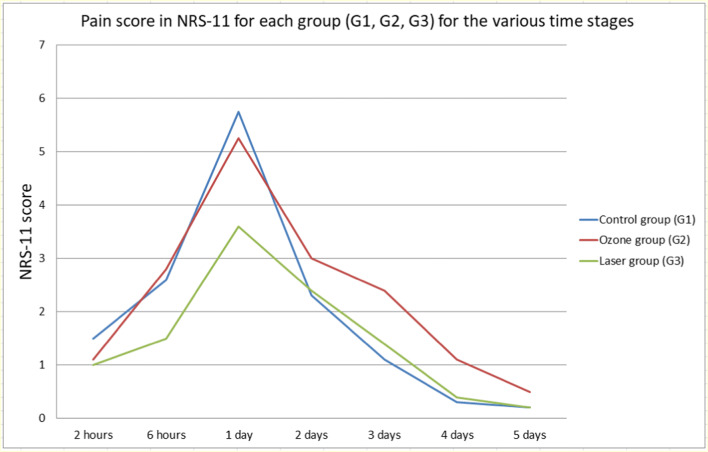
Pain score in NRS-11 for the control (G1), ozone (G2), and laser (G3) group at the different assessment times

Evaluation of pain levels in the control (G1), ozone (G2), and laser (G3) groups of patients at the different assessment times showed that the highest NRS-11 score was 24 h after orthodontic treatment in contrast to various pain assessment times (*p* < 0.05). The abovementioned findings suggested that laser irradiation should be used mainly in the first 24 h post orthodontic appliance placement (Fig. 5).

## Discussion

Pain is a frequent feeling during orthodontic treatment. The purpose of the research was to investigate how photobiomodulation (PBM) and ozone therapy affect the sensation of pain during orthodontic treatment depending on teeth crowding. The main discovery of our study was that patients with crowded teeth and without crowding experience similar discomfort during orthodontic treatment. Furthermore, the laser application was more effective in relieving pain than ozone therapy. The results of this study confirmed that patients experience pain during orthodontic treatment with higher intensity during the first day. In all groups, the pain began 2 h after application of the orthodontic appliance, reaching a maximum after 24 h. Then, it gradually decreased, lasting up to 7 days after the application of orthodontic forces. The average NRS-11 value was 3.60, 5.25, and 5.75 for the diode laser, ozone therapy, and the control group, respectively. The results of our research were confirmed in different studies [3, 8], which found that discomfort reaches its maximum level 24 h after the application of the orthodontic appliance.

In the current research, we applied a diode laser with a wavelength of 635 nm. In the red to the near-infrared spectrum (600–1500 nm), light scattering predominates, and absorption has less effect; thus, the light enters up to a depth of 8–10 mm [27]. The penetration depth of a red laser is lower compared to the infra-red one. However, for the wavelength used in the study (635 nm), the minimum penetration depth is around 3 mm [27]. The aforementioned depth is sufficient to reach the inner part of the soft tissue as well as the tooth apex and the bone. Moreover, the energy dose should be in the range of Arndt-Schultz’s curve; thus, we decided to apply a dose of 2 J per point to reduce the pain after orthodontic appliance placement. LLLT has analgesic, anti-inflammatory, anti-swelling, and regenerative effects. The anti-inflammatory effect is achieved by increasing the secretion of serotonin which induces vasodilation. The concentration of heparin and histamine improves microcirculation and reduces the permeability of blood vessels, which protects against edema [27]. This anti-inflammatory effect is utilized in the management of pain in dental surgery [21, 27], periodontology, temporomandibular joint disorders [28, 29], and orthodontics [23].

One of the main objectives of the current study was to assess whether a diode laser with a wavelength of 635 nm can reduce pain occurring in the first days after orthodontic appliance placement. In our research, we obtained a good result for the laser wavelength at a dose per point of 2 J (400 mW, 4 J/cm^2^), as mentioned above. Similar positive results for a diode laser with a wavelength of 810 nm were shown in studies conducted by Farias at al. [30] (100 mW, 2 J/cm^2^) and Eslamian et al. [31] (100 mW, 2 J/cm^2^). In contrast to the studies mentioned above, there was the research of AlSayed et al. [23], who concluded that a 830-nm diode laser, applied at two doses of 4 and 16 J, was ineffective in relieving orthodontic pain induced by elastomeric separators. Furthermore, in the review published by Li et al. [32], the authors summarized that for the methodological deficiencies and risk of bias of randomized controlled trials, insufficient proof was submitted to conclude whether LLLT was effective in relieving orthodontic pain.

Moreover, positive results of a 940-nm diode laser at 100 mW, 7.5 J/cm^2^ on pain relief during orthodontic treatment were found by Qamruddin et al. [14]. The authors exhibited significantly lower mean NRS-11 score for spontaneous pain after insertion of the initial two archwires (0.012-in and 0.014-in NiTi; *p* < 0.05), while there was no significant difference for 0.016-in and 0.018-in wires between the LLLT and placebo groups [14]. In the present study, we also obtained promising results in pain relief for 0.014-in NiTi archwire after lasing with the 635-nm wavelength, in contrast to the control group. Furthermore, in their study, Bayani et al. [33] compared the effect of NSAIDs, bite wafers, LLLT with two wavelengths (660 nm and 810 nm) in orthodontic pain treatment. It was shown that a laser with a wavelength of 810 nm was the most effective. This finding is relevant because LLLT at various wavelengths could be an alternative to NSAIDs.

A particular focus in the present research was to assess the influence of ozone therapy in orthodontic pain treatment. Our results confirm the null hypothesis of no difference in the pain-reducing effect after the ozone application. The limited effect of ozone can be explained by a too superficial impact on the patient’s tissue, compared to the laser [15]. A further disadvantage of ozone is the decrease in effectiveness when encountering diffusion barriers such as plaque, saliva, and bacterial biofilm. This makes it difficult to penetrate the tissue and thus reduces the effect of ozone. However, ozone is mainly characterized by biocidal activity [15, 16]. During orthodontic treatment, aseptic inflammation of the surrounding tissues is present. Thus, a fundamental feature of ozone is improved oxygenation and nutrition of tissues. However, the use of ozone, which has antiseptic properties, did not lead to a significant reduction in the pain score in our study.

In this present study, we also evaluated the impact of crowding teeth on the sensation of pain during orthodontic treatment. In all the groups studied, there was no statistical difference between the groups explored. Therefore, we can reject the hypothesis that patients with crowded teeth experience greater discomfort than patients with no crowding of teeth. The results of this study confirmed the findings of other researchers who stated that the crowding of teeth or the force exerted on the teeth by the arch do not affect the pain experienced by patients at the beginning of treatment [34, 35]. However, there is no consistent thesis on discomfort during orthodontic treatment. Previous studies by Luppanapornlarp et al. [36] showed that stronger forces applied to the teeth were associated with more severe pain. They tried to compare the intensity of pain associated with exerting different strengths using nickel-titanium coil springs on segmented archwires. According to Hooke’s law, an increase in applied force results in a proportional increase in the deformation of a given material, in this case, an orthodontic wire. The wire can return to its original shape after the cessation of this force. This is known as elastic straining. This property of orthodontic wire is called elasticity. After the insertion of the orthodontic arch in patients with tightly crowded teeth, the orthodontic wire did not behave according to Hooke’s law, exerting the same force regardless of the degree of activation (deformation) [37]. It is expected that the force applied to the teeth will be the same regardless of the degree of tooth crowding. The results of our study can validate this hypothesis.

In our present research, the allocation of subjects to each group was performed randomly by a computer software—Random Allocation Software (University of Medical Science, Isfahan, Iran). However, the age and gender of the patients were not equal in sample size and could contribute to different pain thresholds and thus the overall estimate of the risk of bias for this study was reported to be at medium risk. Moreover, we can expect differences in the pain score in studies where elastic separators or first archwire was applied. Furthermore, there were some other limitations in our present study. We used the NRS scale to rate the feeling of pain, and thus the patients’ assessments were subjective [38, 39]. Therefore, further research should be carried out to search for more objective pain assessment methods during orthodontic treatment.

## Conclusion

There are no differences in pain perception between patients with crowding teeth and non-crowding teeth. The pain was the highest 24 h after orthodontic appliance placement and gradually disappeared in the subsequent 7 days. Our study showed that the best effects in relieving pain were obtained with a laser wavelength of 635 nm. The use of ozone did not have significant effects.
